# Reverse Ratchet Syndrome With Intracardiac Looping

**DOI:** 10.1002/joa3.70489

**Published:** 2026-10-05

**Authors:** Gen Matsuura, Hidehira Fukaya, Tomoharu Yoshizawa, Atsuhiko Sugimoto, Junya Ako

**Affiliations:** ^1^ Department of Cardiovascular Medicine Sagamihara Kyodo Hospital Sagamihara Japan; ^2^ Department of Cardiovascular Medicine Kitasato University Medical Center Kitamoto Saitama Japan; ^3^ Department of Cardiovascular Medicine Kitasato University School of Medicine Sagamihara Kanagawa Japan

**Keywords:** complication, lead displacement, reverse ratchet syndrome, Twiddler syndrome

## Abstract

Intracardiac lead looping developed 7 years after ICD implantation due to reverse Ratchet syndrome. Despite marked radiographic abnormalities, device parameters remained stable and the patient was asymptomatic. Inadequate fixation of the generator and lead sleeves likely contributed to lead advancement. Surgical revision with secure fixation prevented recurrence, highlighting the importance of proper device fixation and long‐term imaging follow‐up to detect delayed lead malposition.

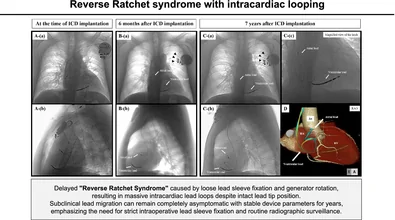

A 59‐year‐old man with dilated cardiomyopathy and non‐sustained ventricular tachycardia underwent implantation of an ICD for primary prevention. The procedure was completed without any complications, and the atrial and ventricular leads were positioned appropriately with stable sensing, pacing thresholds, and lead impedance values. On the post‐implantation chest radiograph, the ICD generator was positioned with the grommet facing caudally (Figure 1A). At the 6‐month routine follow‐up, a chest radiograph revealed that the ICD generator had rotated 180° within the pocket (Figure 1B), whereas the device parameters were unchanged. Seven years later, atrial fibrillation (AF) was detected during routine follow‐up. Chest X‐ray and computed tomography angiography (CTA) showed that the tips of the atrial and ventricular leads remained in their original positions; however, the ICD generator had migrated within the pocket, and both leads had formed large intracardiac loops (Figure 1C,D). The patient was asymptomatic, and no changes were observed in the device parameters.

**FIGURE 1:**
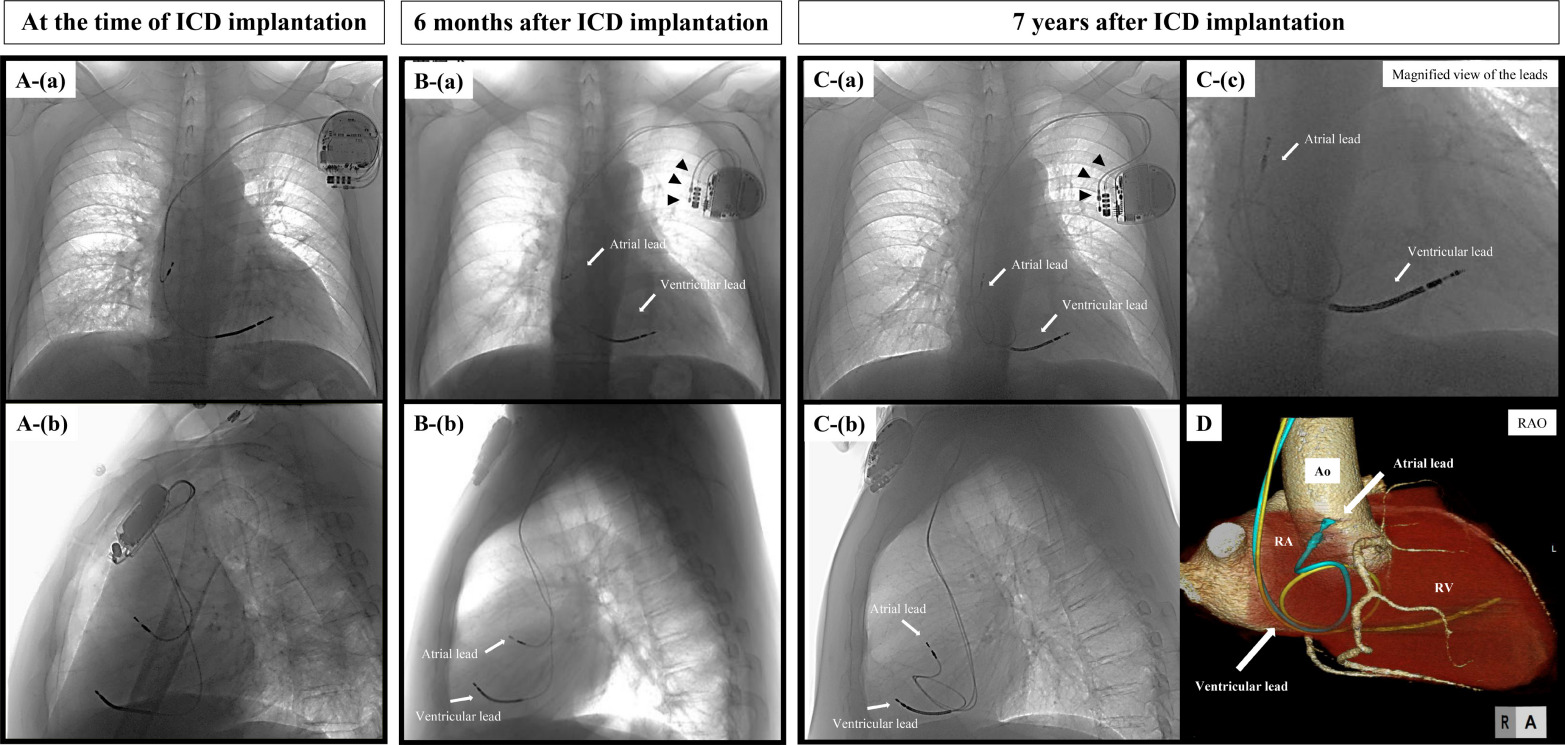
Serial chest X‐rays demonstrating the timeline of ICD generator rotation and lead migration. (A) Chest X‐ray obtained at the time of ICD implantation (a: Posteroanterior, b: lateral view, and c: magnified view of the leads). (B) Chest radiograph obtained 6 months post‐implantation demonstrating a 180° rotation of the generator within the pocket. (C) Chest radiograph obtained 7 years post‐implantation showing marked intracardiac looping of both leads (reverse Ratchet syndrome) while the lead tips remain in their original positions. (D) Computed tomography angiography revealed detailed looping of the intracardiac leads in the atrium and ventricle. Ao, aorta; ICD, implantable cardioverter–defibrillator; RA, right atrium; RV, right ventricle; SVC, superior vena cava.

In addition to catheter ablation for AF, lead repositioning was attempted by simple traction during ICD generator replacement. Intraoperative findings revealed disruption of generator fixation sutures and inadequate ligation of the lead sleeves. The atrial lead loop was successfully released; however, the ventricular lead was adherent to the superior vena cava (SVC), making traction difficult (Figure 2A). The generator and lead sleeves were then securely fixed to the pectoral fascia to prevent further movement. The procedure was completed without any complications, and the patient was discharged in stable condition. No recurrence of AF or lead migration was observed during 6 months of follow‐up (Figure 2B).

**FIGURE 2:**
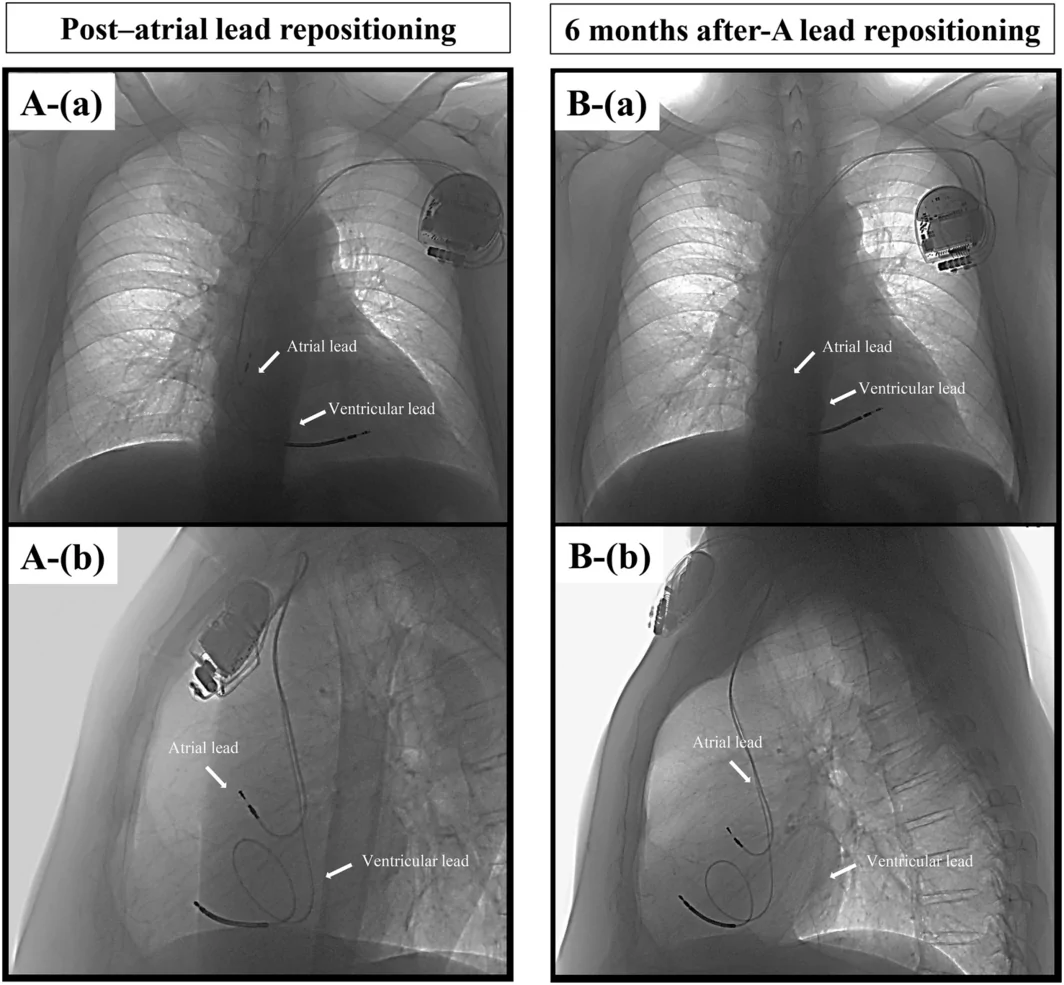
Posteroanterior and lateral chest X‐rays demonstrating temporal changes in leads position post atrial lead repositioning (a, posteroanterior view; b, lateral view). (A) The atrial lead loop was successfully released; however, the ventricular lead was adherent to the SVC, making traction difficult. (B) No lead migration was observed during 6 months of follow‐up. SVC, superior vena cava.

This case highlights a rare but potentially life‐threatening form of lead malposition in CIEDs.

Lead malposition associated with CIEDs is uncommon but clinically important, and several mechanisms have been described [1]. Twiddler [2] and Reel [3] syndromes occur when the device generator rotates within the pocket, resulting in twisting or coiling of the leads, whereas Ratchet syndrome is caused by inadequate fixation of the lead sleeves, allowing gradual lead retraction with repetitive arm movement [4]. Reverse Ratchet syndrome is a rare variant of lead malposition in which the lead progressively advances into the cardiac chamber rather than retracting [5]. This mechanism is thought to occur when the lead sleeve remains insufficiently secured, allowing the lead to progressively advance inward.

This case differs from previous reports in several notable ways. First, cases in which both the atrial and ventricular leads form intracardiac loops are rare, despite the lead tips remaining in their original positions. In the present case, the initial ICD generator had been implanted with the grommet facing caudally. Although the exact operative rationale for this orientation is unknown from the historical records, this inverted positioning may have reduced the mechanical stability of the generator within the pocket. We speculate that this instability facilitated a 180° rotation of the generator once the fixation sutures became disrupted. The combination of generator rotation and loose sleeve fixation likely created an environment that permitted inward lead migration, culminating in reverse Ratchet syndrome. Second, previous reports have described serious complications associated with ventricular leads in reverse Ratchet syndrome, including lead dislodgement [6]. In the present case, however, no lead dislodgement or device malfunction occurred during the 7 years following implantation. One possible explanation is that adhesion of the ventricular lead to the SVC may have contributed to its long‐term stabilization, thereby preventing further advancement or displacement of the lead. Third, despite the occurrence of reverse Ratchet syndrome with inward migration of the leads into the cardiac chamber, simple traction of the ICD lead was difficult. In contrast, the atrial lead loop could be released with gentle traction. This finding further supports the possibility that adhesion of the ventricular lead to the SVC had developed over time, which may have limited further lead movement and contributed to the long‐term stability of the lead tip.

Finally, despite marked radiographic abnormalities, the patient remained asymptomatic and device parameters remained stable. With the increasing number of CIED implantations worldwide, clinicians should be aware that delayed lead malposition can occur even when device parameters remain stable. Careful radiographic evaluation during long‐term follow‐up may help detect such abnormalities. In addition, prevention of lead migration requires an appropriately sized generator pocket, standard cranial placement of the generator, and secure, individual fixation of both the generator and lead sleeves at the time of initial implantation.

In conclusion, reverse Ratchet syndrome may develop even years after CIED implantation and can remain asymptomatic despite marked radiographic abnormalities. These findings highlight the importance of secure fixation of the generator and lead sleeves, as well as careful long‐term follow‐up to prevent and detect delayed lead migration.

## Funding

The authors have nothing to report.

## Ethics Statement

The authors have nothing to report.

## Consent

The authors have nothing to report.

## Conflicts of Interest

The authors declare no conflicts of interest.

## Data Availability

The data that support the findings of this study are available on request from the corresponding author. The data are not publicly available due to privacy or ethical restrictions.
